# Influence of Rootstock on the Leaf Volatile Organic Compounds of Citrus Scion Is More Pronounced after the Infestation with *Diaphorina citri*

**DOI:** 10.3390/plants10112422

**Published:** 2021-11-10

**Authors:** Shelley E. Jones, Nabil Killiny

**Affiliations:** Citrus Research and Education Center, Department of Plant Pathology, IFAS, University of Florida, 700 Experiment Station Road, Lake Alfred, FL 33850, USA; shjones@ufl.edu

**Keywords:** volatile organic compounds, rootstock, scion, citrus, infestation, *Diaphorina* *citri*

## Abstract

Nowadays, citrus greening or Huanglongbing is considered the most destructive disease in the citrus industry worldwide. In the Americas and Asia, the disease is caused by the putative pathogen, ‘*Candidatus* Liberibacter asiaticus’ and transmitted by the psyllid vector, *Diaphorina citri*. It has been shown that volatile organic compounds (VOC) that are released from citrus leaves attract the psyllid vector. Herein, we tested whether the rootstock influenced the stored VOC profile in the scion leaves and if these influences were altered after infestation with *D. citri*. The VOC profiles of the hexane-extracted leaves of the mandarin hybrid ‘Sugar Belle’ that were grafted on three different rootstocks (C-35, sour orange (SO), and US-897) with and without infestation with *D. citri* were studied. The GC-MS analysis showed that the scion VOC profiles of the non-infested control trees were similar to each other, and rootstock was not a strong influence. However, after one month of infestation with *D. citri*, clear differences in the scion VOC profiles appeared that were rootstock dependent. Although the total scion leaf VOC content did not differ between the three rootstocks, the infestation increased scion monoterpenes significantly on US-897 and C-35 rootstock, increased terpene alcohols on US-897 and SO rootstock, and increased sesquiterpenes on SO. Infestation with *D. citri* significantly reduced fatty acids and fatty acid esters across all of the rootstocks. Therefore, our results suggest that rootstock choice could influence scions with an inducible volatile defense by enhancing the amounts of VOCs that are available for repelling vectors or for signaling to their natural enemies or parasitoids. According to this study, US-897 may be the best choice among the three that were studied herein, due to its diverse and robust VOC defense response to infestation with *D. citri*.

## 1. Introduction

Much effort has been given recently to evaluating the disease tolerance of new citrus varieties and scion/rootstock combinations to the citrus disease known as Huanglongbing (HLB) [1,2,3,4,5,6]. Citrus rootstocks are generally selected to improve fruit yield and quality [7,8,9]; help control soil-borne pests and diseases such as *Phytophthora* root rot [10], nematodes [11], and root weevils [12]; defend against abiotic factors [13,14]; or for soil compatibility [8]. Rarely have rootstocks been compared for their contribution to the leaf volatile profile [15,16]. As the leaves are the landing, feeding, and reproduction sites for *Diaphorina citri*, the vector of HLB, determining whether rootstock choice has any influence on leaf volatile organic compounds (VOCs) that could be exploited to repel or deter *D. citri* could be beneficial.

Huanglongbing (HLB), or citrus greening disease, was first discovered in southern China in 1919 [17], and has spread to many citrus growing regions including Brazil and the U.S. [18,19]. In Florida, HLB has been responsible for a decline of 71% in citrus production for the 2016–2017 season compared to 2000–2001 levels [20]. The putative pathogen of HLB is a Gram-positive, phloem-limited α-proteobacteria known as ‘*Candidatus* Liberibacter asiaticus’, which is transmitted by the Asian citrus psyllid, *Diaphorina citri* Kuwayama [Hemiptera: Liviidae] [19,21]. *D. citri* transmits the bacterium from tree to tree while it feeds on the phloem sap of infected citrus trees, and currently, no effective cure is available. The diseased trees produce a low yield of unevenly ripened, lopsided, and bitter fruit, followed by early tree death [22,23]. The fruit from sweet orange trees such as ‘Valencia’ and ‘Hamlin’ are processed into orange juice, and make up the majority of the Florida citrus acreage [24]. Unfortunately, these widely planted varieties are particularly susceptible to HLB disease, with many Florida groves reaching 100% infection [23]. The choice of rootstock can play a role in many horticultural traits such as tree size, fruit yield, and fruit quality [25,26,27,28], as well as tolerance to salinity [29,30]. A recent survey of ‘Hamlin’ on 32 rootstocks in two locations with different soil types showed that the rootstock choice affected the severity of HLB foliar disease symptoms and sturdiness under storm-force winds [31]. However, BowmanandAlbrecht [32] propagated ‘Valencia’ sweet oranges on 11 rootstocks in a greenhouse setting, and found that only a few parameters such as leaf surface area and root susceptibility to ‘*Ca*. L. asiaticus’ were influenced by rootstock. No significant differences were found in the bacterial titer in the leaves after graft-inoculation with ‘*Ca*. L. asiaticus’, and both the leaf size and number were reduced in all of the seedlings after infection regardless of rootstock [32].

Vector control has become the primary weapon for combatting HLB disease, mostly through chemical means. Biological control with the parasitic wasp, *Tamarixia radiata*, has also gained some success [33]. Enhanced citrus nutrition programs to maintain tree productivity have met with mixed results [34], and the development of molecular tools such as interference RNA (RNAi) against *D. citri* are promising, but face practical hurdles outside of the laboratory [35,36]. Finally, many growers have removed heavily diseased trees hoping to replace them with disease-free tolerant varieties, but truly tolerant citrus varieties are still lacking. In 2009, the University of Florida IFAS/CREC cultivar improvement team released many mandarin hybrids including the ‘Sugar Belle’ mandarin hybrid, previously known as LB8-9 [‘Clementine’ mandarin (*Citrus reticulata*) × ‘Minneola’ tangelo (*Citrus* × *Tangelo*), ‘Duncan’ grapefruit (*Citrus paradise*) × ‘Dancy’ tangerine (*Citrus reticulata*)]. Recently published data on the field trials of these new releases suggested that the growth and yield were vigorous in Sugar Belle mandarin trees even in the presence of HLB symptoms, which indicated that it could be tolerant to HLB disease [37]. In greenhouse and semi-field experiments, we found that the Sugar Belle mandarin was high in many secondary metabolites including phenolic compounds, which could act as antibacterial agents, as well as sugar alcohols, which could protect it from stress during pathogen attack [38,39,40]. We found that Sugar Belle was high in several leaf volatiles such as thymol, *β*-elemene, and *β*-caryophyllene, which are known for their anti-microbial activity. These results were obtained from Sugar Belle scions on UF-15 and ‘Carrizo’ rootstocks, respectively [38,39], but these were not comparative studies. Taken together, however, these data indicate that citrus leaf volatiles could play a major role in tolerance against the ‘*Ca*. L. asiaticus’ pathogen.

Among the many roles of leaf volatiles are those of attracting pollinators, repelling herbivores, or attracting their natural predators. Induction of terpenoids as a plant defense following insect herbivory has been widely reported in both annual and perennial crop plants including corn [41], citrus [42], peach [43], and pear [44]. Psyllid-infested pear trees produced more limonene, *α*-farnesene, and *δ*-cadinene after infestation, as well as green leaf volatiles such as (*Z*)-3-hexen-1-yl acetate and (*Z*)-3-hexen-1-ol [44]. Peach trees that were infested with the green peach aphid released higher levels of *β*-ocimene, *α*- and *β*-farnesene, and *E*-nerolidol [43]. Many VOCs also stimulate the volatile defenses of nearby plants (defensive priming) or act as kairomones for attracting the natural predators or parasitoids of the target pest, demonstrating complex inter-trophic relationships between plants and their pests [41]. For example, sour orange trees that were infested with the two-spotted spider mite, *Tetranychus urticae*, showed increased levels of herbivore-induced volatiles including *α*-pinene and 2-ethyl-1-hexanol, and also induced neighboring un-infested ‘Cleopatra’ mandarin trees to produce similar defensive volatile compounds [45]. In rice, the herbivore-induced linalool and *β*-caryophyllene attracted predators and parasitoids of leafhoppers [46], while in citrus, d-limonene and *β*-ocimene attracted the parasitoid of *Thaumatotibia leucotreta*, a pest of citrus in Africa [47].

In our earlier work comparing the leaf volatile organic compounds (VOCs) of 14 citrus varieties [16], we found that citrus cultivars that were considered relatively tolerant to HLB such as Carrizo citrange and *Citrus latipes* contained higher amounts of volatiles than the susceptible cultivars, especially those known for their antimicrobial activities, such as sesquiterpenes and aldehydes. Herein, we hypothesize that rootstock influences the VOC biosynthesis in scion leaves leading to an alteration of the VOCs profile. We expect that this alteration might be more pronounced after the infestation with *D. citri*. To test this hypothesis, we investigated the leaf VOCs of Sugar Belle that was grafted onto three locally available and popular rootstocks, C-35, US-897, and sour orange, with and without infestations of *D. citri*, the vector of HLB.

## 2. Materials and Methods

### 2.1. Plant Materials

Nursery trees of ‘Sugar Belle’, the mandarin hybrid that was previously known as LB8-9, [‘Clementine’ mandarin (*Citrus reticulata*) × ‘Minneola’ tangelo (*Citrus* × *Tangelo*), ‘Duncan’ grapefruit (*Citrus paradise*) × ‘Dancy’ tangerine (*Citrus reticulata*)] that were 9 to 12 months old, were purchased from local citrus nurseries on the rootstocks of the locally available, C-35, sour orange, and US-897. The trees were reared outside, but enclosed in insect-proof, 400-mesh screen cages (1.83 m × 1.83 m × 3.66 m, Bioquip, Rancho Dominguez, CA, USA) at the UF/IFAS Citrus Research and Education Center, Lake Alfred, FL. The Sugar Belle trees were watered three times weekly and fertilized with water soluble fertilizer containing micronutrients once weekly (Peters 20-10-20 Florida Special, ICL Fertilizers, Dublin, OH, USA). The trees were supplemented with Harrell’s (Lakeland, FL, USA) slow-release citrus fertilizer (16-5-10) which also includes minor elements. Trees were confirmed to be ‘*Ca*. L. asiaticus’-free by qPCR as described below.

### 2.2. Asian Citrus Psyllids

The *D*. *citri* that were used for the infestation experiment were reared in our laboratory colony on healthy *Citrus macrophylla* seedlings that were maintained in a climate-controlled growth chamber (27 ± 2 °C, 70 ± 5% relative humidity, 14:10 h L:D photoperiod). This colony was sampled monthly to confirm its ‘*Ca*. L. asiaticus’-free status using quantitative real-time PCR.

#### Infestation with *Diaphorina citri*

Half of the trees from each group were maintained as psyllid-free controls, and half were infested with *D. citri*. To infest the trees, 25 ‘*Ca*. L. asiaticus’ free-psyllids from our laboratory colonies were collected via an insect aspirator into 9-dram snap cap vials (25.2 × 68 mm, #8909, Bioquip, Rancho Dominguez, CA, USA). Each vial of 25 psyllids was placed in a mesh bag that was tied with a ribbon onto the tree branches containing new leaf flush, then the caps were removed to release the psyllids into the bag. The bags were left in place on the branches for one month. After the one-month infestation period, the bags were removed from the branches and the trees were sprayed with 0.1% imidicloprid to kill any remaining adult psyllids or nymphs. A week later, five mature leaves were collected from different locations on each Sugar Belle tree for VOC analysis after the infestation with *D. citri.* The leaves from the control (non-infested) trees were taken at the same time as *D. citri*-infested trees. The leaves were first washed, dried on Kimwipes^®^, and then placed inside zip bags and kept at −30 °C until analysis.

### 2.3. DNA Extraction and qPCR

The DNA extraction from Sugar Belle mandarin trees was accomplished by taking the midribs of the collected leaves (0.1 g fresh weight) and cutting them into small pieces with a clean razor blade. The leaf tissue was placed into 2 mL screw cap tubes and frozen in liquid nitrogen for 10 min. The frozen leaf tissue was processed using a Tissuelyzer II (Qiagen, Germantown, MD, USA) twice at 30 Hz for 1 min each, switching the position of the sample blocks (2 min total). The tubes of the homogenized leaf tissue were removed from the sample blocks and were centrifuged for 1 min at 6000× *g* to pellet the leaf powder. The DNA was obtained using the Plant DNeasy Minikit (#69106, Qiagen, Valencia, CA, USA) following the manufacturer’s protocol.

To confirm that the *D. citri* from our psyllid colony were ‘*Ca*. L. asiaticus’-free, DNA was extracted from single psyllids (10 insects total) using Quick DNA Miniprep Plus kit (#D4068, Zymo Research, Irvine, CA, USA). Briefly, single psyllids were placed into a sterile 1.5 mL centrifuge tube with 95 µL of solid tissue buffer (blue) and 10 µL of proteinase K (provided in the kit), plus 95 µL of water, then homogenized using a hand-held motorized pestle (F65000, BelArt, Wayne, NJ, USA). The homogenate was incubated at 55 °C in a water bath for 2 h. The remaining protocol was followed as given by the manufacturer except that the DNA was eluted into 35 µL of warmed elution buffer to concentrate the DNA.

Real-time quantitative PCR (RT-qPCR) assays were performed on both the plant and insect samples using an Applied Biosystems QuantStudioTM 3 real-time PCR detection system (ThermoFisher Scientific, Waltham, MA, USA) using TaqMan Universal PCR Master Mix (Life Technologies, ThermoFisher Scientific). The dye/quencher set was FAM/NFQ-MGB with carboxyrhodamine (ROX) as the reference dye. the samples were analyzed with the degenerate genus-specific *rpoB* primer/probe set [48]. Each well contained 10 µL TaqMan, 0.6 µL each forward and reverse primers, 0.3 µL *rpoB* probe (both primers and probe from IDT, Coralville, IA, USA), 3.5 µL RNase-free water, and 5 µL DNA template, for a total of 20 µL. The RT-qPCR temperature program consisted of 2 min incubation at 50 °C, 10 min at 95 °C, and 40 cycles at 95 °C for 15 s and 60 °C for 1 min, respectively. The samples were assayed in duplicate and were expressed as the mean cycle threshold (Ct) number. Mean Ct values above 35 or “undetermined” were designated as negative for ‘*Ca*. L. asiaticus’ infection. Pooled DNA from previously tested citrus plants or psyllids with Ct < 25 was used as the plate positive control. All of the plant and insect samples that were used for the experiment tested negative for ‘*Ca*. L. asiaticus’ by RT-qPCR.

### 2.4. Sugar Belle Leaf VOC Extraction

For VOC extraction, a second 0.1 g-aliquot of the same leaf samples (leaf blade portion) was diced, frozen, and homogenized in the same way as mentioned above for DNA extraction. The samples were then placed on ice and were extracted with 0.5 mL hexane by sonication for 5 min, followed by 30 min rotation at 8 °C using an EnviroGenie SI-1200 incubator (Scientific Industries, Bohemia, NY, USA). After centrifuging, 200 µL of the supernatant was transferred to a GC autosampler vial. Each sample was spiked with 2,4-nonadienal as an internal standard (5 µL of 1 mg·mL^−1^), and chromatographic peak areas were normalized to the area of the internal standard.

### 2.5. Analysis of Leaf Volatiles

For each sample, 0.5 µL of leaf hexane extract was injected splitlessly into a Clarus 680-SQ8T GC-MS (Perkin Elmer, Shelton, CT, USA) and the compounds were separated by ZB-5MS column (30 m × 0.25 mm i.d. × 0.25 µm film thickness, Phenomenex, Torrance, CA, USA) using ultrapure helium carrier at 0.9 mL·min^−1^. The oven program was 40 °C for 2 min, ramped to 250 °C at a rate of 7 °C·min^−1^, for a total run time of 32 min, with an initial 5 min solvent delay. The mass spectrometer was running in EI+ mode at 70 eV and scanned from 40–400 amu. The compound peaks were integrated using TurboMass software v.5.4.2 (Perkin Elmer, Waltham, MA, USA) and the mass spectra were compared to those of authentic standards and/or identified by mass spectral libraries (Wiley Flavor and Fragrances of Synthetic and Natural Compounds or NIST 11 (National Institute of Standards and Technology, Gaithersburg, MD, USA) mass spectral databases). The compounds with a library matching score of 700 or better, and with a calculated retention index similar to values in the published literature were considered as tentatively identified. Unidentified VOCs were reported by *m/z* and calculated retention index. The standard reference compounds were purchased from Sigma Aldrich (St. Louis, MO, USA) at the highest purity that was available.

### 2.6. Statistical Analyses

The *p*-values for significance were made at the *p* < 0.05 threshold using the Student’s *t*-test in Microsoft Excel’s Data Analysis ToolPak between infested and non-infested for each Sugar Belle on each rootstock. The variables were assessed for normality using the D’Agostino-Pearson test. The number of replicates for each rootstock was different due to nursery tree availability and were as follows: Sugar Belle/C-35 (12 trees; control *n =* 6, infested *n =* 6); Sugar Belle/US-897 (19 trees; control *n =* 9, infested *n =* 10); and Sugar Belle/Sour Orange (SO) (24 trees; control *n =* 12, infested *n =* 12). The fold changes were calculated by dividing the mean concentration of the *D. citri*-infested samples by that of the control for each compound. The fold changes that could not be calculated due to the absence of one value were designated by an infinity symbol (∞) and signified induction or depletion of that compound after infestation. Principal component analysis, box-and-whisker plots, and the heat map were performed using JMP Pro 16 (SAS Institute, Cary, NC, USA).

## 3. Results

### 3.1. Volatile Organic Compounds Identified in Sugar Belle Leaves

We examined the influence of three rootstocks on the leaf volatile profile of the Sugar Belle mandarin. Table 1 identifies the hexane-extracted volatile leaf metabolites that were detected by GC-MS from Sugar Belle scion leaves that were grown on US-897, C-35, and SO rootstocks, and the groups into which we classified each VOC. A total of 44 volatile organic compounds were extracted from Sugar Belle leaves consisting of 14 monoterpenes; 14 terpene alcohols; 4 sesquiterpenes; 4 fatty acids or their methyl esters (ME); 2 aldehydes; 2 esters, 1 ketone, and coumarin were grouped as “other”; and 2 were unknown compounds (Table 1). Terpene alcohols were the most concentrated, primarily due to linalool. We detected very few terpene aldehydes such as neral and geranial, which are typical of many sweet orange varieties. The overall VOC profile of Sugar Belle mandarins revealed a predominance of *β*-pinene, p-cymene, *β*-ocimene, linalool, *γ*-terpinene, *β*-elemene, and *β*-caryophyllene as the major VOCs.

### 3.2. Principal Component Analysis

**Figure 1:**
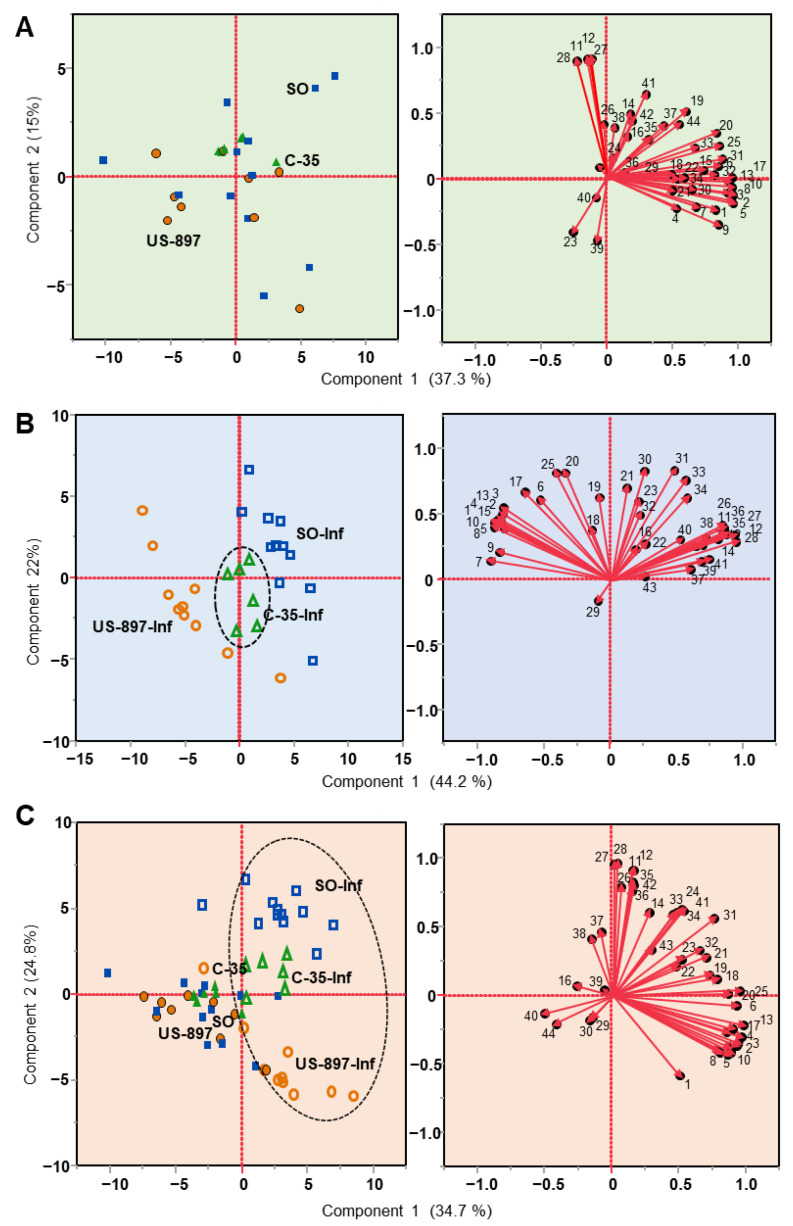
Principal component analysis of the volatile organic compounds (hexane-extracted) from the leaves of the Sugar Belle mandarin hybrid on three different rootstocks (**A**) control trees (non-infested), (**B**) one month after infestation with *Diaphorina citri*, and (**C**) both control trees and *D. citri*-infested trees shown together in the same PCA. The PCA plots show that the Sugar Belle leaf VOC response to *D. citri* was rootstock specific. The numbers in the loading plots correspond to the compounds that are listed in Table 1. PCA symbols: control trees are shown as closed symbols; infested trees are shown as open symbols; US-897 (orange circles); C-35 (green triangles); and SO (blue squares).

To discriminate the effects of rootstock and *D. citri* infestation on the leaf VOC profiles of Sugar Belle, we used principal component analysis (PCA) (Figure 1). qPCR results of citrus plants and infesting *D. citri* showed that both plants and psyllids that were used in the experiments were ‘*Ca*. L. asiaticus’-free. The compound numbers (**1**–**44**) that are found in the loading plots correspond to the numbers and names that are provided in Table 1. The scatter plots represent the VOCs that were present in leaves of the control trees (Figure 1A), *D. citri*-infested trees (Figure 1B), and both the control and infested trees plotted together (Figure 1C). More separation by rootstock is evident in the infested Sugar Belle (Figure 1B) than the non-infested control trees, and when plotted together (Figure 1C), the control trees (closed symbols) clustered together compared to the infested trees (open symbols). Sour orange rootstock grouped in the upper right quadrant of Figure 1A, and the principal components included *α*-pinene, sabinene, *γ*-terpinene, linalool, p-cymene-8-ol, and m-thymol that were found in higher amounts than in the other two rootstocks. The principal components of US-897, which clustered loosely in the lower left quadrant were decanal, thymol methyl ester, and the unknown with *m/z* 69 (Figure 1A). VOCs contributing to the profile of C-35 included *α*-thujene, d-limonene, palmitic acid, methyl linolenate, p-cymenene, *β*-elemene, *β*-caryophyllene, and *α*-humulene. Performing the PCA on the VOC concentrations after *D. citri* infestation provided a much clearer definition of the three rootstocks, demonstrating their differential response (Figure 1B). US-897 clustered across the upper left and lower left quadrants, the sour orange clustered across the upper and lower right quadrants, while C-35 remained clustered in the center (Figure 1B). The compounds contributing to the clustering of US-897 were mainly the monoterpenes (11 monoterpenes were higher in infested US-897 compared to C-35 and SO) and linalool. SO rootstock principal components included nearly all of the terpene alcohols, the two aldehydes, and the sesquiterpenes. C-35 remained clustered in the center with chavicol (#29 in the Figure 1B loading plot, Table 1), *α*-terpinene, and stearic acid as important principal components. Figure 1C shows that the infested rootstocks separated away from each other (in the upper and lower right quadrants) and away from the controls (mostly left of center), indicating a differential response to the infestation with *D. citri*.

### 3.3. Two-Way Hierarchical Cluster Analysis and Heat Map

**Figure 2:**
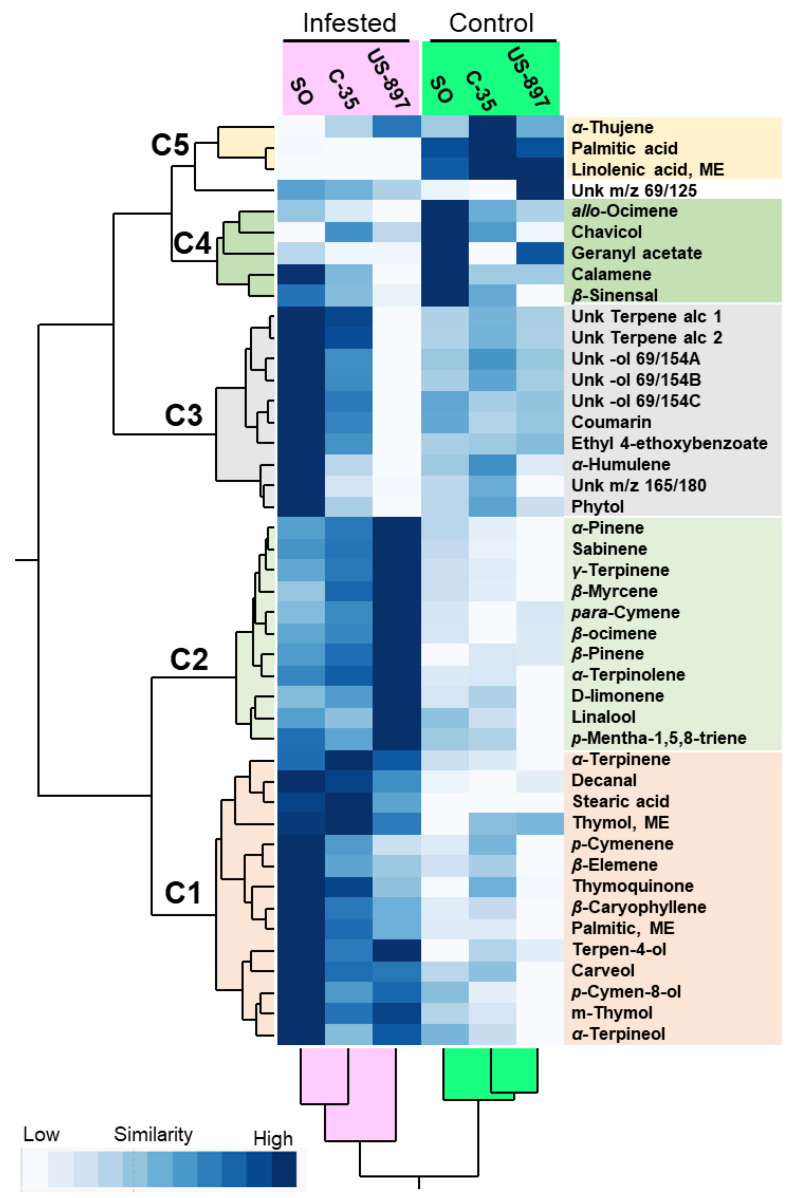
Two-way hierarchical cluster analysis and heat map of VOC concentrations hexane-extracted from the leaves of control and *Diaphorina citri*-infested Sugar Belle trees on three rootstocks (US-897, C-35 trifoliate, and sour orange). The clusters 1–5 represent groups of compounds with a similar response to the infestation for each rootstock. The rows represent the compounds while the columns represent the treatments (control or infested). The cells are the mean concentration (µg·g^−1^) of each compound (*n* ranges from 6 to 12).

A two-way hierarchical cluster analysis and heat map using the means of compounds were generated and are shown in Figure 2. A total of five horizontal clusters were generated using the two-way hierarchical clustering: C1 containing 14 compounds that were associated with infested rootstocks; C2 containing 11 compounds (mostly monoterpenes) that were associated with infested US-897; C3 containing 10 compounds (primarily alcohols) that were associated with infested SO; C4 and C5 containing nine compounds that were associated with control conditions. As expected, the non-infested control samples and the infested samples clustered distinctly in the heat map (Figure 2). In addition, C5 delineated the losses of fatty acids that were found in the infested treatments.

### 3.4. Volatile Profile Alteration after Infestation with Diaphorina citri

We found that the month-long infestation with *D. citri* had a pronounced effect on the stored leaf VOC profiles of Sugar Belle leaves on all of the three rootstocks. Student’s *t*-tests between the control and the infested samples for the individual compounds resulted in significantly different *p*-values (*p* < 0.05) for the majority of the VOCs, which varied by rootstock (Table 2).

For example, almost all of the monoterpenes increased significantly in US-897 and C-35, but about half did not in SO (Table 2). In the SO and US-897, the majority of the alcohols showed significantly different changes in concentration while only about half in the C-35 showed a significant response. The major alcohol, linalool, was higher in all of the infested samples, but only significantly in US-897 (Table 2).

The Sugar Belle leaves from the US-897 rootstock showed the most diverse responses to the infestation with *D. citri.* A total of 16 VOCs decreased (12 significantly, *t*-test *p* < 0.05), while 29 increased (20 significantly) (Table 2). On the other hand, in Sugar Belle leaves that were grown on the C-35 rootstock, 37 compounds increased (22 significantly), while 8 decreased (4 of them significantly). Stearic acid was induced in infested C-35, since it was not detected in the control samples (or was below the limit of detection). SO was similar with 36 VOCs that increased (25 significantly), and 8 decreased (6 significantly) (Table 2).

The concentration of most of the individual compounds increased with infestation consistently between the three rootstocks, as is shown by the fold changes in Table 2. Notable exceptions included *allo*-ocimene (~1.2 to 1.6 fold decreases), and palmitic acid and linolenic acid ME, which both decreased in large amounts in all three of the rootstocks (Table 2). The sesquiterpenes had a mixed response to infestation. *β*-caryophyllene and *β*-elemene increased after the infestation in all of the samples, but in contrast, *α*-humulene and calamene decreased in C-35 and increased in SO. Geranyl acetate, a monoterpene ester, also had a rootstock-dependent response, decreasing significantly in US-897 (*p* < 0.0001) and SO (*p* < 0.0001), but was unchanged in C-35 (*p* = 0.640) (Table 2).

The compounds that increased in concentration by *D. citri* infestation significantly (*p* < 0.05) in all three of the rootstocks included the monoterpenes *β*-pinene, *α*-terpinene, *α*-terpinolene; terpene alcohols *p*-cymene-8-ol, thymol and its methyl ether; thymoquinone, and methyl palmitate (Table 2). Individually, the largest increases in concentration were seen in SO, where terpin-4-ol, thymol methyl ether, and thymoquinone increased more than 3-fold, palmitic acid methyl ester increased 5.9-fold (*p* < 0.0001), and the unknown with *m/z* 69/125 with a retention index of 1749 (possibly 2-octyl-1-dodecanol, library matching score > 850) increased 7.28-fold (*p* < 0.0001). The compounds showing the highest increases on C-35 were palmitic acid ME (4.37-fold, *p* = 0.0006) and the unknown with *m/z* 69/125 (12.69-fold, *p* < 0.0001). Several of the compounds increased more than two-fold in at least one rootstock. These included terpin-4-ol and p-cymene-8-ol in US-897, *α*-terpinene and *α*-terpinolene in US-897 and C-35, unk –ol *m/z* 69/154a in C-35, and *β*-elemene and phytol in SO (Table 2).

### 3.5. VOC Classes Percentage to Each Other (Pie Chart Analysis)

**Figure 3:**
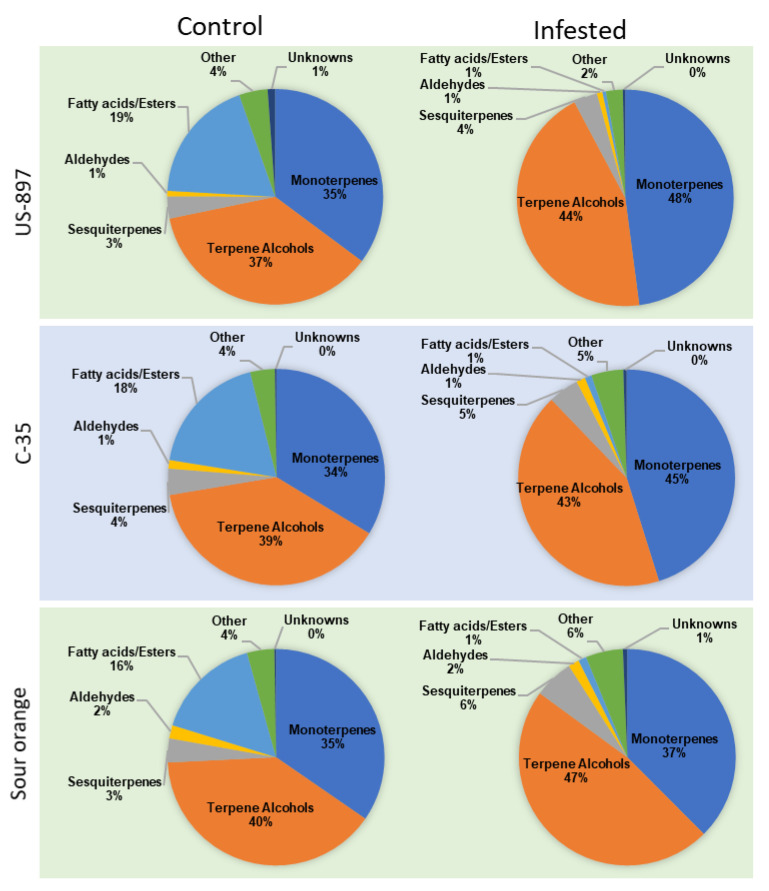
Relative percentages of VOCs by compound class that were detected in the hexane extracts of mature leaves from Sugar Belle mandarin hybrids on three different rootstocks (US-897, C-35 trifoliate, and sour orange), and their changes due to infestation with *Diaphorina citri*.

To visualize the relative changes in VOC composition due to rootstock and infestation, we grouped the compounds by VOC class and calculated percentages of the total concentration (Figure 3). The compounds that were associated with each VOC class can be found in Table 1. We found that in the control trees the composition of Sugar Belle scion leaf volatiles was fairly consistent regardless of the rootstock, consisting of about 35% monoterpenes, 40% terpene alcohols, 18% fatty acids, and 3% sesquiterpenes, 1–2% aldehydes, and with the balance left to a few other compounds and unknowns (Figure 3). After infestation of the Sugar Belle trees with *D. citri*, large decreases in fatty acids were observed in the leaves from all three of the rootstocks and these losses were accompanied by relative increases in monoterpenes in scion leaves from US-897 and C-35 (Figure 3). The gain in monoterpenes was modest in SO (Figure 3). The monoterpene class made up 35.2, 33.7, and 34.6% of the peak area of the control Sugar Belle scion leaf extracts compared to 47.9, 45.2, and 37.5% of extracts from the *D. citri*-infested leaves from US-897, C-35, and SO rootstocks respectively. The monoterpene levels were elevated in *D. citri*-infested samples compared to the controls, and US-897 had both the highest (473.63 µg·g^−1^ F.W.) and lowest (299.13 µg·g^−1^) monoterpene levels in the infested and control samples, respectively. Among the monoterpenes, *γ*-terpinene, *α/β*-pinene, *β*-myrcene, and p-cymene were the most abundant (abundances of all of the compounds can be found in Table 2). Sesquiterpene concentrations increased from 4.6% to 7.49% (*p* < 0.0001) after infestation in scion leaves that were grown on SO compared to C-35 and US-897 (Figure 3).

### 3.6. Concentrations of VOC Classes

**Figure 4:**
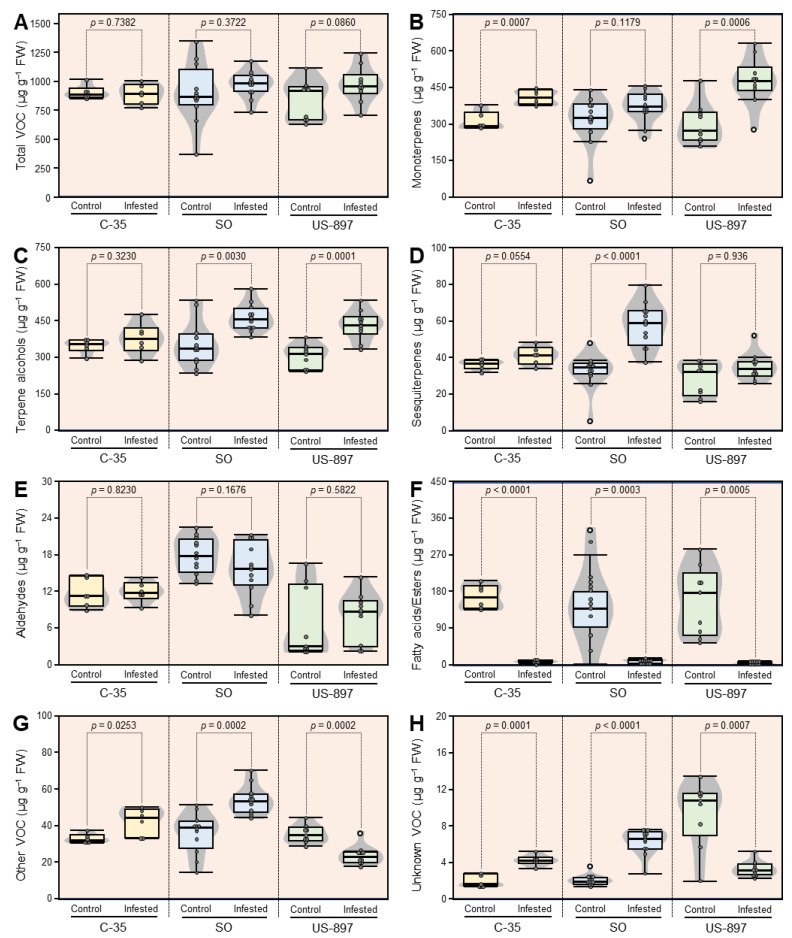
Box plots of the total VOCs levels in each chemical class that were detected in Sugar Belle leaves following infestation with *Diaphorina citri* and the influence of rootstock: (**A**) total VOC content, (**B**) monoterpenes, (**C**) terpene alcohols, (**D**) sesquiterpenes, (**E**) aldehydes, (**F**) fatty acids and their methyl esters, (**G**) other compounds, and (**H**) unknowns. Refer to Table 1 for the VOC classifications. The horizontal thick lines indicate the medians, the black/white dots indicate the means, the boxes show the interquartile ranges including 25–75% of the values, and the whiskers reflect the highest and the lowest value of data. *T*-test *p*-values less than 0.05 are significantly different.

The distribution of the mean concentrations of each VOC class found in Sugar Belle scion leaves that were grown on each rootstock, comparing the non-infested control trees with the *D. citri* infested trees is shown in box-and-whisker plots (Figure 4). The compounds that were associated with each VOC class can be found in Table 1. Interestingly, the total VOCs levels were not significantly different in the scion leaves that were grown on the three rootstocks with or without *D. citri*-infestation (Figure 4A), although shifts in the concentrations within the different VOC classes occurred (Figure 4B–D,F–H). The infestation resulted in moderate alterations in the total VOC content with fold increases of 1.16 and 1.08 for US-897 and SO, respectively, while C-35 total VOCs decreased slightly (−1.02-fold change) compared to the non-infested control trees. The monoterpenes levels were significantly higher in C-35 and US-897 compared to the SO, led by higher levels of *β*-pinene and p-cymene in C-35, and *γ*-terpinene and *β*-ocimene in US-897. In fact, all of the monoterpenes except *allo*-ocimene were higher in US-897 after infestation with *D. citri.* Terpene alcohols increased in both SO and US-897 due to higher levels of thymol and phytol in SO, and linalool and thymol in US-897 (Figure 4C). Sesquiterpenes (Figure 4D) increased significantly (*p* < 0.0001) in the scion leaves that were grown only on SO primarily due to *β*-elemene and *β*-caryophyllene. C-35 sesquiterpenes trended higher after infestation (40.97 µg·g^−1^) compared to 35.73 µg·g^−1^ in the controls (*p* = 0.0554). The fatty acids/esters group (Figure 4F) saw the most dramatic effects of infestation. The total fatty acids in the controls decreased from 169.8 ± 31.1, 144.7 ± 105.5, and 159.2 ± 87.6 µg·g^−1^ in C-35, SO, and US-897, respectively, to 9.3 ± 5.9, 12.1 ± 7.5, and 5.1 ± 3.5 µg·g^−1^, respectively, in the infested samples. Interestingly, the aldehyde levels were higher overall in SO, but were unchanged as a class after infestation (Figure 4E). The group that was designated as other (thymoquinone, ethyl 4-ethoxybenzoate, geranyl acetate, and coumarin) increased significantly in C-35 and SO rootstocks but decreased significantly in US-897 (Figure 4G). In C-35, all four of the compounds increased, three significantly totaling 43.0 ± 7.5, 54.6 ± 7.9, and 24.8 ± 5.65 µg·g^−1^ in C-35, SO, and US-897, respectively, whereas the control levels were 33.7 ± 3.0, 37.0 ± 12.6, and 36.3 ± 5.3 µg·g^−1^, respectively. In SO, thymoquinone increased the most, by 3.57-fold. Geranyl acetate decreased in both US-897 and SO with infection. In US-897, coumarin decreased from 17.5 ± 2.5 µg·g^−1^ to 12.0 ± 2.6 µg·g^−1^ although it increased in C-35 and SO more than 1.3-fold (Figure 4G, Table 2). The mean concentration of the unknowns (Figure 4H) were higher in the infested C-35 and SO, but lower in US-897 with respect to the control means.

## 4. Discussion

Sour orange and *Poncirus trifoliata* “Flying Dragon” are considered “long established” rootstocks in Florida; however, a brief overview of the three rootstocks that were used in this study is provided here. The C-35 rootstock is a “citrange” that is obtained by a cross of ‘Ruby’ sweet orange (*Citrus sinensis* ‘Ruby CRC 589′) with *P. trifoliata*. Its advantages are tolerance to *Phytophthora*, *Citrus tristeza* virus (CTV), drought, and nematodes, but it is sensitive to high salinity and is not good in clay soil (source: https://crec.ifas.ufl.edu/extension/citrus_rootstock, accessed on 15 January 2018). The US-897 rootstock originated from a cross of ‘Cleopatra’ mandarin (*Citrus reticulata* × *P. trifoliata*). It produces compact, short-lived trees (~17 years) with good yield and quality, and shows tolerance to CTV, *Phytophthora palmivora*, and *Diaprepes* root weevil. The sour orange (*Citrus aurantium* L. ‘Sour’) is one of the oldest and most widely used rootstocks for citrus trees. It was commonly planted in Florida until it was found to be susceptible to citrus nematode and CTV, both of which are endemic in Florida [12,49]. Many growers now choose more CTV-tolerant rootstocks (primarily trifoliates), but sour orange (SO) is still available in many Florida nurseries. SO is somewhat tolerant to salinity, alkalinity, and less than optimal drainage, and it is relatively tolerant to the cold and *Phytophthora*. Grapefruit and orange yields on SO are moderate, with average sized, good quality fruit (http://citruspages.free.fr/trifoliates.html#sour, accessed on 15 January 2020).

The contributions of rootstocks toward the HLB tolerance of scion remains controversial. In large field-based studies, researchers have found both profound effects of the rootstock on the scion [4] and little effects on the scion [50]. These mixed results may be due to many factors including grove/tree age, scion type, soil type, differences in nutritional regimes, and delays in HLB symptom development. In an extensive study of the effects of HLB on the yield and fruit quality of ‘Valencia’ sweet orange on multiple rootstocks, Bowman, McCollum and Albrecht [4] found that US-897 had the lowest ‘*Ca*. L. asiaticus’ titer; however, it also had among the lowest yield and fruit size of the many rootstocks that were measured. On the other hand, scion growth on SO and US-897 ranked near the top compared to other rootstocks, and had fewer foliar HLB symptoms in some trials, but not others. In a similar study, symptom severity varied with season [2]. It should be noted that these excellent, multi-year studies were focused on production parameters and overall tree health, rather than the effects of insect infestation on VOC production and serve here to emphasize the many complex variables at play in the citrus response to *D. citri* and HLB.

Rootstock choice can, however, influence the VOC composition of leaves and fruit. In a study of a Pummelo × Grapefruit hybrid on three rootstocks, SO conferred sabinene to the fruit aroma volatiles, while it was absent from fruit that were grown on Volk and *C. macrophylla* rootstock [51]. Both monoterpenes and sesquiterpenes were affected by the rootstock choice, as *β*-elemene and *β*-caryophyllene were significantly higher in fruit grown that were on SO than the other rootstocks [51]. Likewise, the essential oil composition of Persian Lime (*Citrus* × *latifolia*) was affected by rootstock choice, but there was no effect on organoleptic traits such as juice content, pH, titratable acidity, or sugars [52]. Raddatz–Motaetal. [52] found higher levels of aldehydes (desirable in high quality essential oils) in lime oil from fruit grown on the C-35 rootstock than from SO or *P. trifoliata*, and *β*-myrcene was found only in lime oil from fruit that was grown on SO and not from C-35, *P. trifoliata*, Volk lemon, or Swingle rootstocks.

The current study of the Sugar Belle mandarin scion on three different rootstocks found that the extracted VOC content was slightly different in each rootstock before infestation. After infestation, more than 30 of the 44 VOCs were significantly different in the US-897 and SO rootstocks, whereas C-35 had 25 altered VOCs. The dramatic alteration of leaf VOCs after infestation demonstrates that the rootstocks have the potential to contribute to the overall VOC profile of the scion. Interestingly, C-35 and US-897 are trifoliate hybrids of *P. trifoliata* and seedlings of US-897 showed moderate tolerance toward HLB in early comparative studies [53].

Several studies have focused on identifying individual plant volatiles that may repel or attract *D. citri*, for use in lures or as other forms of chemical control [54,55,56,57]. Instead, in this study, we extracted the leaf volatiles of the Sugar Belle mandarin before and after infestation with *D. citri* to identify those that were associated with host response to the infestation. We found that the Sugar Belle scion increased its overall leaf VOC production in response to the infestation regardless of the rootstock onto which it was grafted. The C-35 and SO rootstocks responded similarly, while US-897 gave a stronger and more diverse VOC response to the *D. citri* infestation.

C_10_ monoterpenes derive from C_5_ isoprene units that are catalyzed by terpene synthases, of which more than 50 have been found thus far in the genome of the sweet orange, *Citrus sinensis* [58]. C_10_ monoterpenes are synthesized from geranyl diphosphate (GPP), and C_20_ diterpenes from geranylgeranyl diphosphate (GGPP), both in the methylerythritol phosphate pathway in the plastids [59]. C_15_ sesquiterpenes are synthesized in the mevalonic acid pathway from farnesyl diphosphate (FPP), either in the cytosol and/or in peroxisomes [59,60].

The infestation of Sugar Belle trees with *D. citri* led to increased percentages of monoterpenes (*α*-and *γ*-terpinene, *α*-terpinolene, *β*-ocimene, and p-cymene), sesquiterpenes (*β*-elemene and *β*-caryophyllene), and terpene alcohols (p-cymen-8-ol, thymol, and linalool) in all three of the rootstocks. In our previous study of *D. citri*-infested ‘Valencia’ sweet orange, we found that 14 monoterpenes (C_10_) were elevated by the infestation, led by high levels of *α*-,*β*-pinene, sabinene, *δ*-carene, *β*-ocimene, and *γ*-terpinene [42]. Similar to the current results, total monoterpenes and total sesquiterpenes increased in Tahiti lime fruits that were infested with California red scale, *Aonidiella aurantii* [61]. Individually, the majority of the VOCs increased in concentration with red scale infestation, and five volatiles were produced only by infested fruits (*β*-thujene, limonene oxide, 1-isopropenyl-3-propenylcyclopentane, terpinen-4-ol, and trans-*γ*-bisabolene [61]. Of these five compounds, we detected only terpin-4-ol in increased amounts in *D. citri*-infested trees, but only significantly in the SO rootstock. These differences were perhaps due to the cultivar and tissue type that were analyzed.

In this study, the individual VOCs with the largest induction included the monoterpenes *α*- and *γ*-terpinene, *α*-terpinolene, terpen-4-ol, and thymol methyl ether. These particular monoterpenes and their oxygenated derivatives have well-documented anti-feedant and insect deterrent properties, including deterrence against aphids, mosquitos, and psyllids [62,63,64,65]. In addition, we detected increases in *allo*-ocimene and *β*-ocimene in response to psyllid infestation that was ~1.5-fold higher than in the control trees. Pattetal. [66] detected increased levels of these two monoterpenes in HLB-infected citrus and as a result of priming healthy citrus trees with methyl jasmonate, similar to what would occur during an infestation. We also saw significant increases in *α*- and *β*-pinene, thymol, p-cymene, and p-cymen-8-ol in the psyllid-infested trees. These compounds were larvicidal to mosquito larvae and reduced oviposition of females at concentrations of 20 mg·L^−1^ [67]. On the other hand, *β*-pinene increased the oviposition of female mosquitos and neither *α*-pinene nor *β*-pinene were efficient larvicides without the addition of a cytochrome P_450_ inhibitor [67]. These data suggest that the efficacy of the individual compounds may suffer compared to the natural blends of VOCs that are produced by plants.

Phenylpropanoids and benzenoids, which arise from phenylalanine via phenylalanine ammonia lyase (PAL) to *trans*-cinnamic acid, make up the second largest class of plant VOCs, playing roles in both pollinator mediation and plant defense, and rootstocks can affect changes in the accumulation of these compounds under different abiotic and biotic stresses [9,59]. In this study, we detected several phenylpropanoids and benzenoids, including chavicol, coumarin, and ethyl 4-ethoxybenzoate, and these were consistently reduced in the infested Sugar Belle/US-897, but increased on the C-35 and SO rootstocks. Overall, however, phenylpropanoids and benzenoids represented the smallest portion of leaf VOCs (2–6% of total peak area), as they are more typically associated with floral VOCs [59]. In ‘Moro’ blood orange fruits from C-35 rootstock, the concentrations of chlorogenic, ferulic and sinapic acids were highest among eight rootstocks that were tested by liquid chromatography (HPLC) [26]. The low values that were found in our samples are likely a result of both the tissue type and using GC-MS rather than HPLC, which is more suitable for this class of compounds. For example, in our previous work, Sugar Belle was among the highest of 23 varieties for phenolics and flavonoids content, and Dancy tangerine, one of the parents of Sugar Belle, was highest hydroxycinnamic in acid-related compounds by HPLC methods [68]. Higher levels of these compounds may contribute to the reported tolerance of Sugar Belle toward HLB compared to sweet orange and grapefruit varieties.

Among the highest increased fold changes that were found in Sugar Belle that was infested with *D. citri* were in the fatty acids palmitic acid methyl ester and stearic acid. The roles of fatty acids in plant metabolism are well established and are abundantly present in the leaves, flowers, seeds, and cuticle waxes of many species. The nutritive value of fatty acids that are found in forage or silage crops is critical to the diets of many animals [69], and are valued as essential nutrients for humans. In citrus, fatty acids are found primarily in the peel oil glands, leaves, seeds, and juice sacs (Nordby & Nagy, 1971). Gueta–Dahanetal. [70] correlated a 20% increase in palmitic acid (C16:0) and linoleic acid (C18:2) content with abiotic stress (salt stress) in a salt-tolerant cultured citrus cell line, and with an accompanying decrease of 50% in linolenic acid (C18:3) content. The changes in lipid metabolism were mediated constitutively by superoxide dismutase (SOD) in an effort to quench reactive oxygen species [70]. Interestingly, we found the opposite effect in psyllid-infested citrus trees, suggesting that biotic stress may alter citrus lipid metabolism through a different mechanism.

The infestation with *D. citri* initiated a severe decline in the levels of methyl linolenate. One possible explanation for this decline could be that α-linolenic acid, the precursor of jasmonic acid (JA), is released from plastid membranes as a defense against both phytophagous insects and pathogen attack [62,64,65]. Jasmonic acid is found in very low abundance in plant material, requiring a specific extraction using acidified methanol and ethyl acetate, concentration under nitrogen gas, and sometimes derivatization prior to GC-MS analysis, usually in the selected ion (SIM) mode [71,72]. We recently described the response of citrus to the “double attack” of *D. citri* and “*Ca*. Liberibacter asiaticus” on citrus phytohormones [72]. Although *D. citri* are not traditional chewing insects, the infestation likely induced a cascade of responses initiating the biosynthesis of JA. Since we did not extract or analyze the leaf VOCs in a manner that would optimize the detection of JA in the same samples, it is not possible to determine if the final metabolic product was, in fact, JA.

In addition to fatty acid-derived plant signals, insects also utilize fatty acids, which serve as precursors for many specialized features of insects such as flight, sex and alarm pheromones, wax production, and cold adaptation (overwintering). Fatty acids are critical to the development and metamorphosis of many insects, and lipids may be acquired during feeding from plant hosts directly, or by carbohydrate conversion in the fat body [73]. For example, linolenic acid methyl ester was required for the physiological development in Lepidopterans [74,75] and in *Drosophila* [76]. Palmitic acid was the dominant fatty acid in the cornicle secretions of the brown citrus aphid, *Aphis* (*Toxoptera*) *citricida*, at 86.5 mM [77].

Fatty acid esters are also components of the leaf cuticle (wax) [78,79]. An alteration of leaf cuticle composition by the presence of infesting insects has been reported. In sorghum, leaf surface microscopy revealed changes in the pigments and lipid structure after aphid feeding [80]. The changes in lipid composition of cutin were reported in wheat after Hessian fly (*Mayetiola destructor*) infestation [81], and in olive fruit surfaces after olive fly egg-laying [34]. Changes in leaf thickness and mineral content were reported in citrus leaves after citrus leaf miner infestation, but there was no FA analysis of the leaf cuticle [53]. In corn plants, aphid-stressed seedlings induced an increase in maldondialdehyde (a lipid oxidation biomarker) [82]. Again, our extraction was non-specific for triglycerides and fatty acids, and our results may not adequately represent the true composition of lipids compared to other methods. However, we can hypothesize that fluctuations in fatty acid content of the Sugar Belle leaves were the result of either the direct utilization by *D. citri* during the course of the infestation, an indirect response to the infestation by the host plant, or both.

## 5. Conclusions

With the epidemic spread of HLB in the United States, as well as in many citrus producing countries, there is an urgent need to provide growers with commercial citrus cultivars that are more tolerant to HLB, such as the Sugar Belle mandarin. Current field trials that are evaluating HLB tolerance mostly rely on already infected trees or new plantings that are exposed randomly to field populations of *D. citri*. Controlling the exposure to psyllids in a greenhouse or in semi-field conditions could help elucidate and optimize the plant defense response of many rootstock/scion combinations. Identifying those combinations with the strongest defense response against *D. citri* can then be field-tested. The evidence found thus far suggests that the scions with an inducible volatile defense perform better against infestations by releasing VOCs which directly repel phytophagous insects or which signal to their natural enemies or parasitoids. According to this study, US-897 may be the best choice among the three rootstocks that were studied herein due to its diverse and robust VOC defense response to infestation with *D. citri*.

## Figures and Tables

**Table 1:** Volatile organic compounds (VOCs) that were detected by GC-MS in the hexane extracts of mature leaves from Sugar Belle mandarin hybrid. Compound numbers are given for their association with Figure 1 PCA loading plots.

Compound No.	Leaf VOC	VOC Class	Compound No.	Leaf VOC	VOC Class
**1**	*α*-Thujene	Monoterpene	**23**	Thymol, ME	Terpene alcohol
**2**	*α*-Pinene	Monoterpene	**24**	Thymoquinone	Other
**3**	Sabinene	Monoterpene	**25**	m-Thymol	Terpene alcohol
**4**	*β*-Pinene	Monoterpene	**26**	Unk -ol *m*/*z* 69/154A	Terpene alcohol
**5**	*β*-Myrcene	Monoterpene	**27**	Unk -ol *m*/*z* 69/154B	Terpene alcohol
**6**	*α*-Terpinene	Monoterpene	**28**	Unk -ol *m*/*z* 69/154C	Terpene alcohol
**7**	p-Cymene	Monoterpene	**29**	Chavicol	Terpene alcohol
**8**	D-limonene	Monoterpene	**30**	Geranyl acetate	Monoterpene ester
**9**	*β*-ocimene	Monoterpene	**31**	*β*-Elemene	Sesquiterpene
**10**	*γ*-Terpinene	Monoterpene	**32**	*β*-Caryophyllene	Sesquiterpene
**11**	Unk Terpene alc 1	Terpene alcohol	**33**	α-Humulene	Sesquiterpene
**12**	Unk Terpene alc 2	Terpene alcohol	**34**	Unk *m*/*z* 165/180	Unknown
**13**	*α*-Terpinolene	Monoterpene	**35**	Coumarin	Other
**14**	p-Cymenene	Monoterpene	**36**	Ethyl 4-ethoxybenzoate	Other
**15**	Linalool	Terpene alcohol	**37**	Calamene	Sesquiterpene
**16**	*allo*-Ocimene	Monoterpene	**38**	*β*-Sinensal	Aldehyde
**17**	p-Mentha-1,5,8-triene	Monoterpene	**39**	Unk *m*/*z* 69/125	Unknown
**18**	Terpen-4-ol	Terpene alcohol	**40**	Palmitic acid	Fatty acid
**19**	p-Cymen-8-ol	Terpene alcohol	**41**	Palmitic, ME	Fatty acid ester
**20**	*α*-Terpineol	Terpene alcohol	**42**	Phytol	Terpene alcohol
**21**	Decanal	Aldehyde	**43**	Stearic acid	Fatty acid
**22**	Carveol	Terpene alcohol	**44**	Linolenic acid, ME	Fatty acid ester

**Table 2:** The volatile metabolites from hexane-extracted leaves of the Sugar Belle (SB) mandarin hybrid on three different rootstocks comparing the non-infested control and Asian citrus psyllid (ACP)-infested trees (mean ± standard deviation of the mean). The concentration units are µg·g^−1^ FW based on 100 mg fresh weight of leaves that were extracted with 0.5 mL hexane. The quantification was made by using calibration equations that were obtained by plotting the peak area vs. concentration of each reference standard. The standards were serially diluted in hexane and injected into the GC-MS in the same volume and under the same conditions as the leaf extracts.

Cmpd No.	LRI	Leaf VOC	SB/US-897				SB/C-35				SB/Sour Orange			
			Control	ACP-Infested	*p* Value	Fold Change	Control	ACP-Infested	*p* Value	Fold Change	Control	ACP-Infested	*p* Value	Fold Change
**1**	900	*α*-Thujene ^b^	22.03 ± 7.06	23.80 ± 6.08	0.566	1.08	25.98 ± 2.84	20.62 ± 1.62	**0.002**	−1.26	21.03 ± 5.44	18.40 ± 3.52	0.173	−1.14
**2**	909	*α*-Pinene ^a^	29.46 ± 9.43	49.77 ± 12.71	**0.001**	1.69	31.01 ± 4.01	43.06 ± 3.29	**0.000**	1.39	34.30 ± 12.22	39.44 ± 7.25	0.224	1.15
**3**	962	Sabinene ^a^	2.88 ± 0.87	4.58 ± 1.13	**0.002**	1.59	2.97 ± 0.45	4.06 ± 0.32	**0.001**	1.37	3.25 ± 1.09	3.82 ± 0.67	0.139	1.17
**4**	966	*β*-Pinene ^a^	26.25 ± 8.25	45.44 ± 11.42	**0.001**	1.73	26.56 ± 3.84	39.37 ± 2.77	**0.000**	1.48	23.85 ± 14.36	34.92 ± 6.86	**0.025**	1.46
**5**	985	*β*-Myrcene ^a^	8.91 ± 2.90	12.84 ± 3.12	**0.011**	1.44	9.25 ± 1.25	11.82 ± 0.86	**0.002**	1.28	9.56 ± 3.09	10.11 ± 2.10	0.620	1.06
**6**	1018	*α*-Terpinene ^a^	1.87 ± 1.12	4.64 ± 2.24	**0.004**	2.49	2.30 ± 0.55	5.16 ± 0.75	**0.000**	2.24	2.59 ± 1.64	4.37 ± 1.18	**0.006**	1.69
**7**	1026	p-Cymene ^a^	38.37 ± 8.88	66.19 ± 10.64	**0.000**	1.72	34.81 ± 4.73	51.83 ± 6.33	**0.000**	1.49	38.48 ± 10.83	44.50 ± 9.73	0.166	1.16
**8**	1034	D-limonene ^a^	14.41 ± 4.47	21.56 ± 5.33	**0.006**	1.50	16.04 ± 3.33	17.61 ± 1.34	0.311	1.10	15.24 ± 6.12	16.61 ± 3.02	0.494	1.09
**9**	1055	*β*-ocimene ^a^	41.10 ± 17.25	69.27 ± 14.30	**0.001**	1.69	38.01 ± 9.09	56.24 ± 7.14	**0.003**	1.48	41.76 ± 13.97	50.99 ± 10.09	0.077	1.22
**10**	1071	*γ*-Terpinene ^a^	93.61 ± 24.92	145.50 ± 32.00	**0.001**	1.55	98.47 ± 12.75	127.78 ± 9.42	**0.001**	1.30	102.45 ± 33.20	115.82 ± 19.63	0.243	1.13
**11**	1094	Terpene alc 1 ^c^	3.60 ± 0.47	2.56 ± 0.38	**0.000**	−1.41	4.01 ± 0.21	5.32 ± 0.18	**0.000**	1.32	3.56 ± 0.88	5.56 ± 0.31	**0.000**	1.56
**12**	1099	Terpene alc 2 ^c^	2.21 ± 0.28	1.58 ± 0.22	**0.000**	−1.40	2.49 ± 0.11	3.25 ± 0.11	**0.000**	1.30	2.19 ± 0.55	3.45 ± 0.17	**0.000**	1.58
**13**	1101	*α*-Terpinolene ^a^	7.67 ± 3.02	15.96 ± 4.58	**0.000**	2.08	8.84 ± 1.37	14.36 ± 1.10	**0.000**	1.62	8.68 ± 3.61	12.94 ± 2.42	**0.003**	1.49
**14**	1110	p-Cymenene ^b^	9.58 ± 1.22	10.19 ± 2.71	0.543	1.06	10.90 ± 1.04	11.46 ± 2.10	0.569	1.05	9.95 ± 3.32	13.83 ± 2.27	**0.003**	1.39
**15**	1114	Linalool ^a^	184.35 ± 35.17	296.81 ± 68.44	**0.000**	1.61	202.80 ± 22.99	219.40 ± 47.27	0.457	1.08	218.88 ± 76.07	235.18 ± 40.42	0.519	1.07
**16**	1160	*allo*-Ocimene ^b^	0.74 ± 0.21	0.61 ± 0.16	**0.168**	−1.20	0.81 ± 0.18	0.67 ± 0.15	0.179	−1.21	1.22 ± 0.48	0.76 ± 0.23	**0.006**	−1.61
**17**	1172	p-Mentha-1,5,8-triene ^b^	2.29 ± 0.68	3.27 ± 0.74	**0.008**	1.43	2.60 ± 0.49	2.82 ± 0.31	0.384	1.08	2.66 ± 0.92	3.03 ± 0.54	0.237	1.14
**18**	1225	Terpen-4-ol ^a^	0.44 ± 0.52	1.16 ± 0.18	**0.001**	2.66	0.61 ± 0.48	0.93 ± 0.24	0.165	1.54	0.34 ± 0.26	1.16 ± 0.25	**0.000**	3.39
**19**	1224	p-Cymen-8-ol ^a^	0.29 ± 0.21	0.70 ± 0.20	**0.000**	2.41	0.34 ± 0.17	0.59 ± 0.21	**0.047**	1.75	0.50 ± 0.46	0.81 ± 0.23	**0.050**	1.62
**20**	1236	*α*-Terpineol ^a^	2.50 ± 0.59	3.69 ± 0.97	**0.006**	1.47	2.84 ± 0.58	3.12 ± 0.58	0.426	1.10	3.16 ± 0.78	3.90 ± 0.78	**0.030**	1.23
**21**	1243	Decanal ^a^	2.64 ± 1.07	3.23 ± 0.58	0.147	1.22	2.54 ± 0.24	3.62 ± 0.47	**0.001**	1.42	2.59 ± 0.87	3.71 ± 0.80	**0.004**	1.43
**22**	1259	Carveol ^a^	0.29 ± 0.32	0.47 ± 0.19	0.150	1.62	0.40 ± 0.13	0.48 ± 0.26	0.537	1.19	0.36 ± 0.26	0.53 ± 0.25	0.127	1.45
**23**	1268	Thymol, Me ^a^	0.64 ± 0.12	0.77 ± 0.09	**0.012**	1.21	0.61 ± 0.05	0.90 ± 0.10	**0.000**	1.49	0.23 ± 0.30	0.89 ± 0.11	**0.000**	3.91
**24**	1250	Thymoquinone ^b^	0.28 ± 0.09	0.52 ± 0.11	**0.000**	1.86	0.57 ± 0.27	0.88 ± 0.15	**0.033**	1.55	0.26 ± 0.09	0.94 ± 0.17	**0.000**	3.57
**25**	1324	m-Thymol ^a^	65.70 ± 12.55	95.31 ± 14.30	**0.000**	1.45	71.27 ± 7.29	89.72 ± 8.48	**0.002**	1.26	75.99 ± 18.96	97.42 ± 15.94	**0.007**	1.28
**26**	1336	Unk -ol 69/154A ^c^	3.29 ± 2.63	ND	**NA**	∞	2.86 ± 3.16	5.84 ± 0.83	**0.049**	2.04	4.37 ± 1.75	8.22 ± 1.29	**0.000**	1.88
**27**	1342	Unk -ol 69/154B ^c^	4.03 ± 0.77	2.39 ± 0.83	**0.000**	−1.69	4.91 ± 0.32	5.05 ± 0.48	0.585	1.03	3.99 ± 1.16	6.51 ± 0.42	**0.000**	1.63
**28**	1347	Unk -ol 69/154C ^c^	2.47 ± 0.50	1.51 ± 0.61	**0.002**	−1.63	2.96 ± 0.05	3.27 ± 0.37	0.064	1.11	2.42 ± 0.74	4.33 ± 0.52	**0.000**	1.79
**29**	1378	Chavicol ^a^	1.40 ± 0.61	1.53 ± 0.68	**0.659**	1.10	1.74 ± 0.34	1.78 ± 0.13	0.806	1.02	2.13 ± 0.80	1.38 ± 0.37	**0.007**	−1.55
**30**	1385	Geranyl acetate ^a^	1.35 ± 0.49	0.45 ± 0.11	**0.000**	−3.01	0.42 ± 0.07	0.45 ± 0.13	0.640	1.07	1.55 ± 0.58	0.65 ± 0.14	**0.000**	−2.40
**31**	1396	*β*-Elemene ^a^	12.04 ± 3.95	16.50 ± 4.09	**0.028**	1.37	16.12 ± 1.25	18.91 ± 2.90	0.056	1.17	14.40 ± 4.95	29.46 ± 5.17	**0.000**	2.05
**32**	1461	*β*-Caryophyllene ^a^	10.78 ± 3.79	13.90 ± 2.72	0.053	1.29	12.38 ± 1.01	16.10 ± 1.91	**0.002**	1.30	11.52 ± 4.05	18.90 ± 7.90	**0.009**	1.64
**33**	1512	*α*-Humulene ^a^	4.20 ± 1.81	3.85 ± 0.89	0.586	−1.09	6.01 ± 0.58	4.67 ± 0.68	**0.004**	−1.29	4.93 ± 1.22	7.99 ± 1.63	**0.000**	1.62
**34**	1525	Unk *m*/*z* 165/180 ^c^	1.59 ± 0.49	1.61 ± 0.37	0.954	1.01	1.96 ± 0.31	1.71 ± 0.35	0.232	−1.14	1.79 ± 0.60	3.01 ± 0.75	**0.000**	1.69
**35**	1534	Coumarin ^b^	17.47 ± 2.50	11.98 ± 2.63	**0.000**	−1.46	16.27 ± 1.29	21.67 ± 3.79	**0.008**	1.33	19.26 ± 3.88	26.77 ± 4.11	**0.000**	1.39
**36**	1554	Ethyl 4-ethoxybenzoate ^b^	17.18 ± 2.21	11.86 ± 2.78	**0.000**	−1.45	16.42 ± 1.33	19.97 ± 3.43	**0.039**	1.22	15.96 ± 8.07	26.26 ± 3.51	**0.001**	1.65
**37**	1724	Calamene ^b^	1.22 ± 0.26	0.91 ± 0.31	**0.031**	−1.35	1.22 ± 0.11	1.29 ± 0.32	0.608	1.06	1.68 ± 0.37	1.67 ± 0.82	0.974	−1.01
**38**	1734	*β*-Sinensal ^a^	4.23 ± 6.40	4.94 ± 3.86	0.767	1.17	9.28 ± 2.38	8.47 ± 1.30	0.481	−1.10	15.40 ± 2.96	12.14 ± 4.42	**0.045**	−1.27
**39**	1746	Unk m/z 69/125 ^c^	8.01 ± 3.41	1.89 ± 0.65	**0.000**	−4.24	0.21 ± 0.33	2.69 ± 0.40	**0.000**	12.69	0.47 ± 0.12	3.39 ± 1.12	**0.000**	7.28
**40**	1967	Palmitic acid ^a^	62.38 ± 32.71	0.19 ± 0.22	**0.000**	−329.73	72.34 ± 7.33	0.18 ± 0.43	**0.000**	−409.03	63.44 ± 46.08	0.95 ± 0.66	**0.000**	−66.80
**41**	2000	Palmitic, ME ^a^	0.71 ± 0.49	3.69 ± 1.30	**0.000**	5.15	1.45 ± 0.31	6.34 ± 2.39	**0.001**	4.37	1.46 ± 2.57	8.62 ± 2.91	**0.000**	5.90
**42**	2075	Phytol ^a^	39.69 ± 16.43	29.36 ± 30.49	0.379	−1.35	56.24 ± 12.13	44.53 ± 17.15	0.202	−1.26	42.51 ± 19.75	98.62 ± 26.98	**0.000**	2.32
**43**	2088	Stearic acid ^a^	0.41 ± 1.22	1.24 ± 1.98	0.292	3.06	ND	2.75 ± 3.07	NA	∞	1.34 ± 2.23	2.51 ± 3.89	0.374	1.88
**44**	2094	Linolenic acid, ME ^a^	95.70 ± 53.18	ND	NA	∞	95.96 ± 23.47	ND	NA	∞	78.46 ± 54.65	ND	NA	∞

^a^ Compound confirmed by authentic reference standard. ^b^ Tentatively identified by LRI and library matching score > 700. ^c^ Unknown compound present consistently in samples and containing ion fragments typical of known citrus VOCs. Bold *p* values indicate significance (*p* < 0.05). Fold change was calculated by dividing the average of *D. citri*-infested by control.
